# Surgical management and long-term outcome of intracranial subependymoma

**DOI:** 10.1007/s00701-018-3570-4

**Published:** 2018-06-18

**Authors:** Adithya Varma, David Giraldi, Samantha Mills, Andrew R. Brodbelt, Michael D. Jenkinson

**Affiliations:** 1 School of Medicine, University of Liverpool, Cedar House, Ashton Street, Liverpool, L3 5PS UK; 2 Department of Neurosurgery, The Walton Centre NHS Foundation Trust, Liverpool, UK; 3 Department of Neuroradiology, The Walton Centre NHS Foundation Trust, Liverpool, UK; 4 Institute of Translational Medicine, University of Liverpool, Liverpool, UK

**Keywords:** Adults, Intracranial subependymoma, Neurosurgery, Surgical outcome, WHO performance status

## Abstract

**Background:**

Intracranial subependymomas account for 0.2–0.7% of central nervous system tumours and are classified as World Health Organization (WHO) grade 1 tumours. They are typically located within the ventricular system and are detected incidentally or with symptoms of hydrocephalus. Due to paucity of studies exploring this tumour type, the objective was to determine the medium- to long-term outcome of intracranial subependymoma treated by surgical resection.

**Methods:**

Retrospective case note review of adults with intracranial WHO grade 1 subependymoma diagnosed between 1990 and 2015 at the Walton Centre NHS Foundation Trust was undertaken. Tumour location, extent of resection (defined as gross total resection (GTR), sub-total resection (STR) or biopsy) and the WHO performance status at presentation and through follow-up were recorded.

**Results:**

Thirteen patients (7 males; 6 females) with a mean age of 47.6 years (range 33–58 years) and a median follow-up of 46 months (range 25–220 months) were studied. Eight patients had symptomatic tumours (headache, visual disturbance); five had incidental finding. Tumours were most commonly located in the fourth ventricle (*n* = 8). The performance status scores at diagnosis were 0 (*n* = 8) and 1 (*n* = 5). The early post-operative performance status scores at 6 months were 0 (*n* = 5) and 1 (*n* = 8) and at last follow-up were 0 (*n* = 11) and 1 (*n* = 2). There was no evidence of tumour re-growth following GTR or STR. The commonest complication was hydrocephalus (*n* = 3).

**Conclusion:**

Subependymoma are indolent tumours. No patients exhibited a worsening of performance status at medium- to long-term follow-up and there were no tumour recurrence suggesting a shorter follow-up time may be sufficient. Surgical resection is indicated for symptomatic tumours or those without a clear imaging diagnosis. Incidental intraventricular subependymoma can be managed conservatively through MRI surveillance.

## Introduction

Subependymomas are rare, indolent neoplasms that are histologically classified as low grade (World Health Organization (WHO) grade 1 [11]) and represents only 0.2–0.7% of all central nervous system tumours [8, 14, 16]. They present in both sexes and all age groups, but occur most frequently in the middle-aged to older individuals, typically in the fifth and sixth decade of life [10]. The majority of cases are asymptomatic but symptoms caused by hydrocephalus or spontaneous tumour haemorrhage may occur for larger tumours in the fourth, lateral and third ventricles, septum pellucidum and, less commonly, the spinal cord [1, 11, 14, 19]. Despite its benign nature, there are several reports of tumour recurrence and metastasis within the central nervous system following surgical resection [6, 9, 14].

Compared to other intracranial tumours, there is a relative paucity of published series on subependymoma focusing on long-term post-operative outcomes. As a result, the duration of follow-up required for these patients is not clear. A better understanding of the long-term outcomes would not only benefit patients but also reduce health care costs. The aims of this study were to determine the medium- to long-term outcome of intracranial subependymoma treated by surgical resection and also and to discuss the change in paradigm of subependymoma management.

## Methods

### Study population

Adult patients with a histological diagnosis of subependymoma (WHO grade 1) between January 1990 and December 2015 at The Walton Centre NHS Foundation Trust were included in the study. Patients with cranial ependymoma, subependymal giant cell astrocytoma and spinal cord tumours were excluded from this study. Clinicopathological data were obtained and analysed through retrospective case note review, outpatient follow-up and serial magnetic resonance imaging (MRI) scans.

Tumour location (defined as left lateral ventricle (LLV), right lateral ventricle (RLV), third or fourth ventricle), the surgical approach, extent of resection (defined as biopsy, sub-total resection (STR) or gross total resection (GTR) based on the operative notes and post-operative MRI), post-surgical complications and WHO performance status (Table 1) pre-surgery and until final follow-up were assessed.

**Table 1:** Description of World Health Organization performance status scores

Grade	Explanation of activity
0	Fully active, able to carry on all pre-disease performance without restriction
1	Restricted in physically strenuous activity but ambulatory and able to carry out work of a light or sedentary nature, e.g. light house work, office work
2	Ambulatory and capable of all self-care but unable to carry out any work activities. Up and about more than 50% of waking hours
3	Capable of only limited self-care, confined to bed or chair more than 50% of waking hours
4	Completely disabled. Cannot carry on any self-care. Totally confined to bed or chair
5	Dead

### Image analysis

**Fig. 1:**
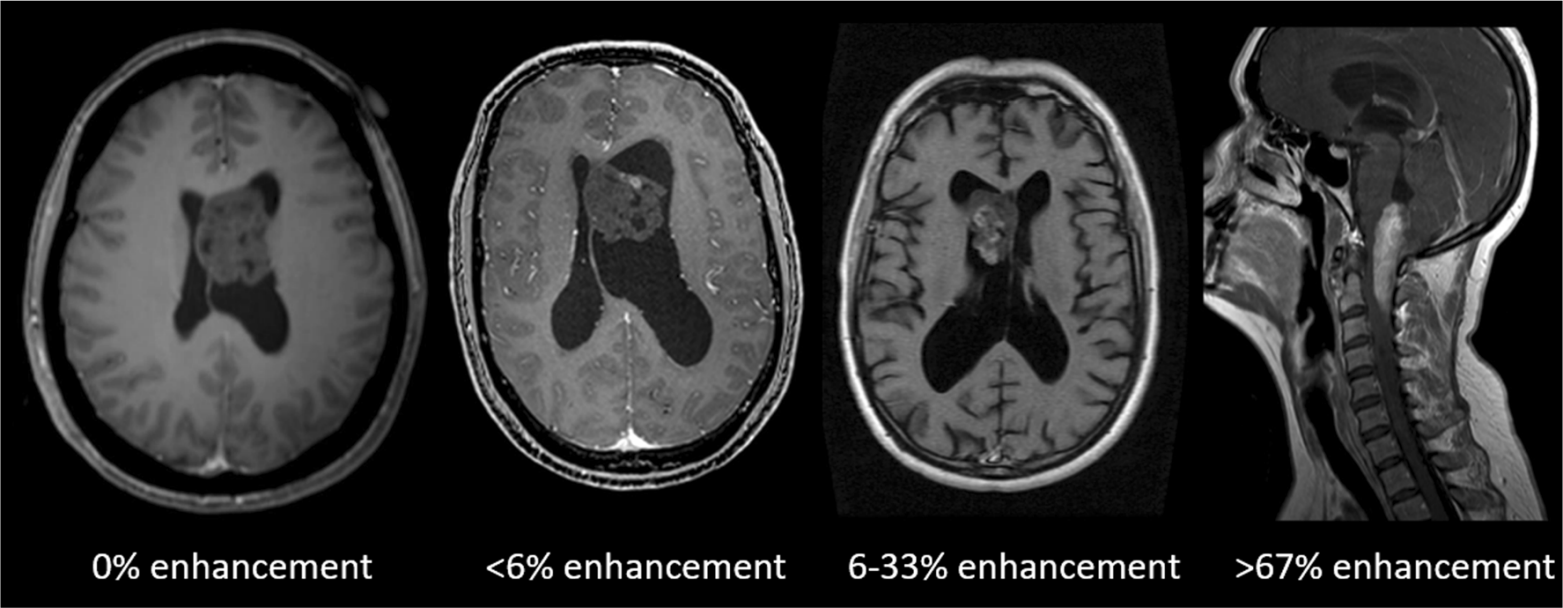
Representative case examples of varying degrees of proportional contrast enhancement on post-gadolinium contrast T1-weighted imaging

Where available, pre-operative MRI studies were assessed for lesion signal intensity characteristics on T1 and T2 sequences relative to grey matter, binary assessment of the presence or absence of enhancement following administration of contrast agent, categorical assessment of the proportion of contrast enhancement if present (< 6%, 6–33%, 34–67% and > 67%; shown in Fig. 1) and two-dimensional measures of maximum dimensions in three planes.

### Statistical analysis

Descriptive statistical analysis was performed using SPSS Statistics v.22 Chicago, II.

## Results

### Clinical presentation

Thirteen patients (seven males and six females) with histologically proven intraventricular subependymomas were identified (clinical data summarised in Table 2). The age ranged from 33 to 58 years (mean 47.6 years). The most common presenting symptoms were headaches (*n* = 7) and blurred vision (*n* = 5). The tumour was an incidental finding in five cases; the rationale for surgery was due to patient choice or diagnostic uncertainty about tumour type. Further breakdown of the patients with incidental findings are stated in Table 2.

**Table 2:** Clinical features of 13 subependymoma patients in the study

Case no.	Sex/age (years)	Symptoms	Tumour location	Hydrocephalus at presentation	Operative approach	Degree of resection	WHO performance status score (0–5)						Follow-up (months)
							Pre-op	Post-op	6 months	12 months	24 months	Last follow-up	
1	M/56	Headache, blurred vision, altered sensation in left hand	RLV	Y	Interhemispheric transcallosal approach for craniotomy	GTR	1	1	1	0	0	0	220
2	F/51	Headache, diplopia, loss of balance	LLV and 3rd ventricle	N	Interhemispheric transcallosal approach for craniotomy	GTR	0	1	1	0	0	0	84
3	F/33	*Incidental finding*—new onset epilepsy. MRI revealed 4th ventricular tumour	4th ventricle	N	Linear occipital incision for posterior fossa craniotomy	GTR	0	1	0	0	0	0	91
4	F/50	Seizure with incontinence, feeling faint, blurring of vision	LLV	Y	Interhemispheric transcallosal approach for craniotomy	GTR	0	1	1	0	0	0	85
5	M/36	*Incidental finding*—CT for head injury following dog attack. Further MRI showed tumour	RLV	N	Interhemispheric transcallosal approach for craniotomy	GTR	0	1	0	0	0	0	46
6	M/52	*Incidental finding*—review 2 years following thoracotomy for thoracic Schwannoma, CT scan showed lesion supratentorially	4th ventricle	N^a^	Linear posterior fossa incision for cranitomy	GTR	0	1	1	0	0	0	50
7	M/47	*Incidental finding*—patient was diagnosed with symptomatic Chiari malformation. MRI showed incidental 4th ventricular tumour	4th ventricle	N	Linear occipital incision for Chiari decompression with tumour resection	GTR with Chiari decompression	0	1	1	0	0	0	54
8	M/50	Headache, blurred vision, short-term memory loss	LLV	N	Interhemispheric transcallosal approach for craniotomy	GTR	1	1	0	0	0	0	37
9	M/55	Headaches	4th ventricle	N	Linear midline sub-occipital incision for craniectomy	GTR	0	1	0	0	0	0	31
10	F/35	Blurred vision, loss of balance, numbness and weakness in arms	4th ventricle	N	Linear midline sub-occipital incision for craniectomy	GTR	1	1	1	0	0	0	32
11	F/54	Loss of balance, blurring of vision	4th ventricle	Y^a^	Linear midline sub-occipital incision for craniectomy	GTR	1	1	1	1	1	1	28
12	M/58	*Incidental finding*—MRI of cervical spine for investigation of brachalgia. Incidental 4th ventricle tumour found	4th ventricle	N^a^	Linear midline posterior fossa incision for craniectomy	STR- tumour stuck to vertebral artery	0	1	0	0	0	0	35
13	F/42	Headache, weakness in arms and short-term memory loss	4th ventricle	N	Linear occipital incision for craniotomy	GTR	1	1	1	1	1	1	25

^a^Evidence of post-operative hydrocephalus

### MRI findings

Eight tumours were located in the fourth ventricle (61.5%), two in LLV (23.1%) and two in the RLV (15.4%) and in one patient, the tumour was present in both LLV and third ventricle. Three had radiologic evidence of hydrocephalus at presentation.

Pre-operative MRI scans were available for analysis in nine patients (Table 3). T1-weighted scans showed low to intermediate signal intensity subependymoma and T2-weighted scans mainly exhibited hyperintense tumours in relation to the brain parenchyma. Contrast enhancement was observed in eight of the nine tumours, with half demonstrating minimal enhancement (< 6%). No presence of calcification was noted in any MRI scans.

**Table 3:** Radiologic features of the 13 patients in the study

					Tumour size (mm)		
Case number	T1 signal intensity	T2 signal intensity	Contrast enhancement	Size of enhancing area	Anterior-posterior	Left-right	Cranio-caudal
4	Low to intermediate	Hyperintense	Non-enhancing	N/A	38	29	29
5	Low to intermediate	Hyperintense	Enhancing	6–33%	35	30	33
6	Low	Hyperintense	Enhancing	6–33%	19	15	25
7^b^	N/A	N/A	N/A	N/A	N/A	N/A	N/A
8	Low	Hyperintense	Enhancing	< 6%	32	36	40
9	Intermediate	Intermediate	Enhancing	< 6%	20	15	20
10	Low to intermediate	Intermediate	Enhancing	> 67%	20	25	68
11	Intermediate	Hyperintense	Enhancing	34–67%	13	14	15
12	Intermediate	Hyperintense	Enhancing	< 6%	12	16	17
13	Intermediate	Hyperintense	Enhancing	< 6%	11	16	19

^b^Tumour not radiologically visible as found incidentally during Chiari decompression

### Extent of resection

For supratentorial subependymoma, all five patients underwent an interhemispheric transcallosal approach for GTR of the tumour. For the remaining eight patients with infratentorial subependymoma (fourth ventricle), a standard sub-occipital craniectomy or craniotomy was used. The surgical approaches used were discussed at the multi-disciplinary meetings and were in concordance with the operating surgeon’s preference. Endoscopy was not used for any cases. In this group of eight cases, GTR was achieved in seven patients and STR in one patient due to tumour adherence to the vertebral artery. This group also included one patient who underwent a GTR of the incidental subependymoma when operated for Chiari I malformation. The overall gross total resection was achieved in 12 patients (92.3%) and STR in one patient (7.7%).

### Complications

Post-operative complications included cerebral spinal fluid leak (*n* = 1) that resolved with a period of temporary lumbar drainage and hydrocephalus (*n* = 3), requiring treatment with ventriculoperitoneal shunt (*n* = 2) or endoscopic third ventriculostomy (*n* = 1). Only one of the three patients with post-operative hydrocephalus had pre-operative hydrocephalus. No patients developed a central nervous system infection or any new neurological deficits following surgery. One patient who was noted to have reduced cardiac output suffered from a cardiac arrest post-operatively but recovered sufficiently to be discharged home after 3 weeks. That patient was noted to have memory deficit and impaired cognition that subsequently improved but did not return to his pre-morbid state.

### Performance status and clinical outcomes

The median length of follow-up was 46 months (range 25–220 months). Patients underwent annual MRI and clinical assessment. No patients received any adjuvant oncological therapy. There were no cases of tumour recurrence or re-growth in either the GTR or STR groups.

WHO performance status score from diagnosis until last follow-up for all patients is summarised in Table 2. The performance status at diagnosis was 0 (*n* = 8) and 1 (*n* = 5) and at the early post-operative period at 6 months was 0 (*n* = 5) and 1 (*n* = 8). Four patients had a sustained decline in WHO performances status score from 0 to 1 at 6 months whilst one patient had an improvement in performances status from 1 to 0 during this time period. At last follow-up, the performance status score was 0 (*n* = 11) and 1 (*n* = 2).

Overall, the WHO performance status score improved in three patients (23.1%) [GTR *n* = 3] at last recorded follow-up compared to the performance status score at the time of diagnosis, whereas in ten patients (76.9%) [GTR *n* = 8, STR n = 1], it remained unchanged.

Two out of three patients with post-operative hydrocephalus showed an improvement in performance status by 12 months following treatment, the other patient exhibiting a stable performances status following treatment.

## Discussion

Intraventricular subependymomas are rare tumours and most neurosurgeons will only operate on a small number of cases during their career. To date, there is sparse reporting of post-operative outcomes for patients following tumour removal due to its low incidence [1, 6, 9, 13]. Surgery for subependymoma is usually reserved for symptomatic individuals who usually present with hydrocephalus [1, 6, 9]. In our study, regardless of the extent of resection there were no cases of tumour recurrence and all patients had WHO performance status of 0 or 1 at last follow-up.

Whilst the Karnofsky performance status (KPS) measures functional outcome on a ten-point scale, it has largely been replaced in neuro-oncology studies with the WHO performance status. In our study, the WHO performance status at the latest follow-up improved in 23% (*n* = 3) and stayed the same in 77% (*n* = 10) compared to pre-operatively. Performance status was unchanged after 24 months for all the patients and none reported worsening of performance status at their last outpatient appointment. In addition, no patients received additional oncological therapy and there were also no cases of tumour recurrence.

In a series of 11 cases [9], good post-operative outcomes were observed with a median KPS of 90 at last follow-up (range 60–100) with 91% (10/11) of patients reporting a higher or equivalent KPS score compared to their pre-operative performance status [9]. Similar outcomes were reported in a series of 26 cases with 92% of patients (24/26) having KPS that were higher or equivalent to pre-operative values [6]. There was no tumour recurrence noted in either study [6, 9]. Combining the data from both series, only 4/37 patients had a worsening of performance status at last follow-up, all of whom underwent a GTR. Whilst these data are limited, they suggest that STR may be a reasonable surgical option in patients where GTR is not possible since there was no statistically significant difference in survival based on the extent of resection [12, 15]. In our study, only one patient underwent STR and although there was no recurrence, it is possible that with longer follow-up recurrence may yet occur. Previous studies have shown evidence of tumour progression 6 years following STR in one patient that was managed with a second operation without any subsequent complications at a further 7 years follow-up [9]. The variation in tumour behaviour and risk of recurrence suggests that long-term follow-up is needed for patients treated with STR.

In contrast to these findings, tumour recurrence was reported in another series involving 43 patients [1]. This cohort consisted of 34 patients with pure subependymoma (27 adults and 7 children), 8 mixed with ependymoma and 1 mixed with astrocytoma. Of those with pure subependymoma, only 3% (1/34) experienced recurrence of their tumour [1]. Nearly 25% of this group of patients, mainly children, underwent STR with no reported cases of recurrence. Regarding tumour recurrence, this study theorised that poorly defined borders were an independent predictor of shorter progression-free survival (PFS) with subependymoma [1] and may lead to tumour recurrence in patients. Despite a handful of cases across several studies, recurrence of subependymoma is considered a rare event [6, 8, 9, 18]. The extended follow-up period of patients emphasises this finding—the longest follow-up duration in the respective studies were 115 months [9] (age 48 at diagnosis) and 188 months [6] (age 44 at diagnosis) and one patient from our study was followed up for 220 months (aged 56 at diagnosis), none of whom demonstrated any evidence of tumour re-growth. These suggest a possible need for earlier discharge of patients, especially those with GTR and no post-operative hydrocephalus or ventriculoperitoneal shunts.

Radiologically, subependymomas are typically well demarcated, non-enhancing, nodular lesions [12, 17] usually in the fourth ventricle [1, 9, 15]. On pre-contrast MRI studies, they are generally hypo- or isointense to grey matter in T1 signal intensity and hyperintense on T2 modality [12, 17], and this was observed in our series. We noted contrast enhancement in 89% of our cohort which is not typical for subependymoma, but rather is more frequently seen in ependymoma [2]. However, the degree of enhancement was minimal, especially compared to ependymoma where contrast enhancement is more florid. There were too few cases in our study to undertake any analysis of MRI features to predict the likelihood of complete resection or to develop post-operative hydrocephalus. In our cohort, hydrocephalus was the most common complication (*n* = 3; 23%) following subependymoma resection with patients requiring treatment with ventriculoperitoneal shunt within 1 month (*n* = 2) or an endoscopic third ventriculostomy (*n* = 1). This rate is higher than previously reported (7–19% [1, 6, 9]), but the patients in our cohort did not show any long-term clinical deterioration or consequences from their hydrocephalus and had a stable WHO performance status throughout follow-up (see Table 2). This highlights the successful treatment of hydrocephalus with relatively low morbidity [1, 6, 9].

## Historical perspective

**Fig. 2:**
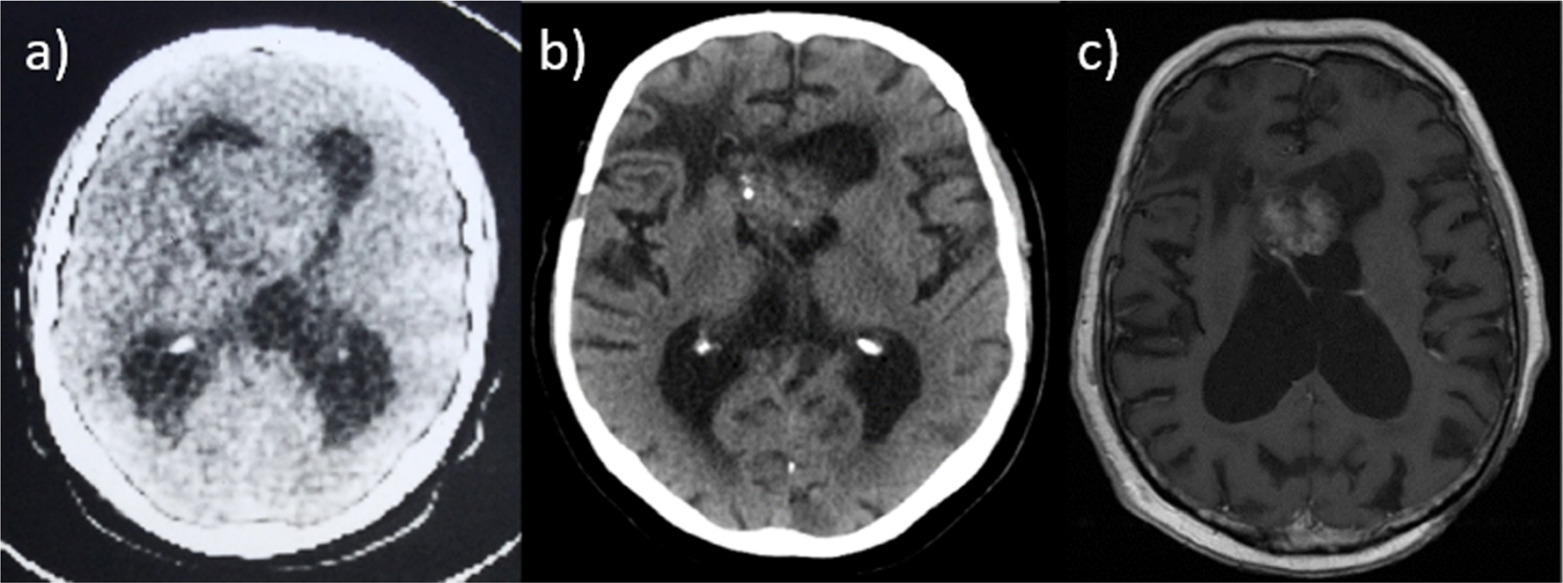
**a** CT scan of lateral ventricle subependymoma (taken in 1979). More recent imaging (CT (**b**) and MRI axial T1 post-gadolinium contrast, (**c**)) demonstrating stability of the subependymoma size over the long surveillance period

Historically, the pathophysiology and natural history of subependymoma was less well understood and there has been a paradigm shift in the management of these tumours over time [16, 18]. This is exemplified in a case from our neurosurgical archives which revealed a patient who presented with raised intracranial pressure headache in 1979 and a cranial computer tomography (CT) showing a large intraventricular tumour (Fig. 2a). Diagnosis of subependymoma was made following craniotomy and open biopsy. Surgical resection was not considered due to the potential risk of morbidity and the patient was treated with craniospinal irradiation. The patient was discharged after 5-year follow-up but re-presented to the neurosurgical service with headache 36 years after surgery. CT and MRI (Fig. 2b, c) show no tumour growth or progression but revealed several radiation-induced meningiomas. Clinically, the patient suffered pituitary dysfunction and cognitive decline as late effects of radiotherapy. This case highlights the extremely indolent nature of subependymoma supporting the findings of this series.

Evolved microneurosurgical techniques aided by neuronavigation ensure an efficient and safer approach for maximal tumour excision [5, 14]. These methods have also led to a significant reduction in complication rates from 23.5–33% in the 1990s [13, 15, 16] to 0–15% in more recent surgical series [1, 4, 8, 9, 13, 18]. In hindsight, the radiation therapy received by our patient was injurious and is no longer part of modern management of subependymoma. In contrast, stereotactic radiosurgery has been used successfully for small diameter subependymoma [3, 7] with no tumour recurrence reported in a case following 54 months of follow-up [3]. Whether this could be considered as an alternative first-line treatment in subependymoma remains to be determined due to the lack of reported cases.

This was a retrospective review of surgically treated cases of an uncommon intracranial tumour. It should be noted that only 13 patients were involved in our study which is a small sample size but to our knowledge, this is the only study to explore the effects of subependymoma resection on the WHO performance status over such a long follow-up period. Surgery is established as the treatment of choice for symptomatic subependymoma and the medium- to long-term decline in performance status has not been noted in our series. The imaging features in our series were slightly atypical for subependymomas with eight out of nine cases showing some contrast enhancement and this is likely to have contributed to the decision to offer surgery to these patients—the clinical concern being that these tumours could be ependymoma.

## Study limitation

This was a single-institution retrospective study that carries the inherent risks of bias. Nevertheless, this adds to the number of published series on subependymoma that enables the opportunity to perform a meta-analysis to better understand the risk of tumour recurrence and complication rates. A large multi-centre study is unlikely to occur in view of the extended follow-up required. Although we were able to derive the performance status from case notes, a more detailed assessment of quality of life was not available. In addition, for patients undergoing resection of lateral ventricle tumours, there was no formal assessment of cognitive function and memory which can be affected following surgery via an interhemispheric transcallosal approach. Nevertheless, patients had a good performance status, which supports the notion that there was no appreciable neurological harm.

## Conclusion

Subependymomas are rare, benign tumours. No recurrence or deterioration in WHO performance status was noted during follow-up in our cohort suggesting a shorter follow-up time. Tumour resection is required for symptomatic tumours or where there is radiological uncertainty about the diagnosis and good neurological outcomes can be achieved with microneurosurgery. Incidental intraventricular subependymomas can be managed conservatively through MRI surveillance thereby avoiding the need for surgery.

## Abbreviations

WHO: World Health Organization
MRI: Magnetic resonance imaging
LLV: Left lateral ventricle
RLV: Right lateral ventricle
STR: Sub-total resection
GTR: Gross total resection
KPS: The Karnofsky performance status
PFS: Progression-free survival
CT: Computer tomography
